# Supplementary material for: National adaptation and implementation of WHO Model List of Essential Medicines: A qualitative evidence synthesis
Source: PLoS Med. 2022 Mar 11;19(3):e1003944. doi: 10.1371/journal.pmed.1003944 (PMC8956172; doi:10.1371/journal.pmed.1003944)
Supplement: S1 Text — (DOCX) [file pmed.1003944.s002.docx]

**S1 text: List of full texts reviewed and reasons for exclusion**

| **No.** | **Title** | **Authors** | **Year** | **Journal** | **Volume** | **Issue** | **Reason for exclusion** |
| --- | --- | --- | --- | --- | --- | --- | --- |
| 1 | Evidence-based decision-making in Asia-Pacific with rapidly changing health-care systems: Thailand, South Korea, and Taiwan | Jirawattanapisal, T. K., P.; Lee, T. J.; Yang, M. C. | 2009 | Value Health | 12 | Suppl 3 | Does not include original qualitative data |
| 2 | [Evaluation of pharmaceutical assistance in public primary care in Brasilia, Brazil] | Naves Jde, O. S., L. D. | 2005 | Rev Saude Publica | 39 | 2 | Does not include original qualitative data |
| 3 | National essential medicines list of Pakistan: Use in prescriptions and awareness in medical practitioners | Tahir, M.; Irfan, M. | 2017 | Journal of Postgraduate Medical Institute | 31 | 3 | Does not include original qualitative data |
| 4 | A public-health approach to site-specific formulary management: Addressing deficient drug supplies in Malawi | Lauffenburger, J. C.; Jonkman, L. J.; Lange, C. E.; Connor, S. E.; Sibale, C. | 2011 | International Journal of Pharmacy Practice | 19 | 3 | Does not include original qualitative data |
| 5 | Drug policy in Nicaragua, between need-oriented activities and aggression | Laporte, J. R. T., G. | 1985 | Dev Dialogue |  | 2 | Does not include original qualitative data |
| 6 | The essential drugs in Tanzania: a ten year (1979-1988) review following the Alma Ata declaration | Kasilo, O. J. M., G. M. | 1992 | East Afr Med J | 69 | 5 | Does not include original qualitative data |
| 7 | An evaluation of the Essential Medicines List, Standard Treatment Guidelines and prescribing restrictions, as an integrated strategy to enhance quality, efficacy and safety of and improve access to essential medicines in Papua New Guinea | Joshua, I. B.; Passmore, P. R.; Sunderland, B. V. | 2016 | Health Policy & Planning | 31 | 4 | Does not include original qualitative data |
| 8 | Essential medicines and human rights: What can they learn from each other? | Hogerzeil, H. V. | 2006 | Bulletin of the World Health Organization | 84 | 5 | Does not include original qualitative data |
| 9 | WHO Model List of Essential Medicines and developed countries: A comparison with the Lothian Joint Formulary | Hems, S.; Laing, R. | 2006 | WHO Drug Information | 20 | 2 | Does not include original qualitative data |
| 10 | An analysis of China&#039;s national essential medicines policy | Guan, Xiaodong; Liang, Huigang; Xue, Yajiong; Shi, Luwen | 2011 | Journal of Public Health Policy | 32 | 3 | Does not include original qualitative data |
| 11 | Efficacy of constitutional support to enhance access to essential medicines as a human right to health in the Eastern Mediterranean Region | Elsayed, L. M. | 2012 | Eastern Mediterranean Health Journal | 18 | 1 | Does not include original qualitative data |
| 12 | Availability and rational use of drugs in primary healthcare facilities following the national drug policy of 1982: is Bangladesh on right track? | Ahmed, S. M. I., Q. S. | 2012 | J Health Popul Nutr | 30 | 1 | Does not include original qualitative data |
| 13 | Artemisinin and its derivatives: the regulatory and policy implications for African countries | Salako, L. | 1998 | Med Trop (Mars) | 58 | Suppl 3 | Does not include original qualitative data |
| 14 | Essential medicines availability is still suboptimal in many countries: a scoping review | Mahmic-Kaknjo, M.; Jelicic-Kadic, A.; Utrobicic, A.; Chan, K.; Bero, L.; Marusic, A. | 2018 | Journal of Clinical Epidemiology | 98 |  | Does not include original qualitative data |
| 15 | Recommendations of the Expert Committee for the Selection and inclusion of Medicines in the PAHO’s Strategic Fund 2015. Washington | Pan American Health, O. | 2016 | Generic |  |  | Does not include original qualitative data |
| 16 | Assessment of core drug use indicators using WHO/INRUD methodology at primary healthcare centers in Bahawalpur, Pakistan | Atif, M. S., M. R.; Azeem, M.; Naz, M.; Amir, S.; Nazir, K. | 2016 | BMC Health Serv Res | 16 | 1 | Does not include original qualitative data |
| 17 | Availability of essential medicines in selected public, primary and secondary health care institutions of a rural Sri Lankan district: a spot survey | Rathish, D. P., I.; Jayathilake, T.; Kandegedara, C.; Punchihewa, K.; Ananda, L.; Bandara, T.; Jayasumana, C.; Siribaddana, S. | 2017 | BMC Health Serv Res | 17 | 1 | Does not include original qualitative data |
| 18 | Essential drugs and registration of pharmaceuticals: the Sri Lankan experience | Weerasuriya, K. | 1993 | Bull World Health Organ | 71 | 2 | Does not include original qualitative data |
| 19 | Comparative analysis of essential medicines for cardiovascular diseases in countries of the WHO Eastern Mediterranean Region | Mehrtash, H. L., Richard; Wirtz, Veronika J. | 2018 | Eastern Mediterranean Health Journal | 24 | 5 | Does not include original qualitative data |
| 20 | Availability of essential medicines in primary health care of the Brazilian Unified Health System | Nascimento, R. A., J.; Guerra, A. A. Junior; Gomes, I. C.; Costa, E. A.; Leite, S. N.; Costa, K. S.; Soeiro, O. M.; Guibu, I. A.; Karnikowski, M. G. O.; Acurcio, F. A. | 2017 | Rev Saude Publica | 51 | suppl 2 | Does not include original qualitative data |
| 21 | Availability of essential drugs for managing HIV-related pain and symptoms within 120 PEPFAR-funded health facilities in East Africa: a cross-sectional survey with onsite verification | Harding, Richard; Simms, Victoria; Penfold, Suzanne; Downing, Julia; Higginson, Irene J. | 2014 | Palliat Med | 28 | 4 | Does not include original qualitative data |
| 22 | A new entity for the negotiation of public procurement prices for patented medicines in Mexico | Gomez-Dantes, O. W., V. J.; Reich, M. R.; Terrazas, P.; Ortiz, M. | 2012 | Bull World Health Organ | 90 | 10 | Does not include original qualitative data |
| 23 | Assessment of drug use patterns in terms of the WHO patient-care and facility indicators at four hospitals in Southern Ethiopia: a cross-sectional study | Gidebo, K. D. S., T. S.; Kanche, Z. Z.; Woticha, E. W. | 2016 | BMC Health Serv Res | 16 | 1 | Does not include original qualitative data |
| 24 | Effectiveness of pharmacy interventions in improving availability of essential medicines at the primary healthcare level | Nunan, M. D., T. | 2011 | Trop Med Int Health | 16 | 5 | Does not include original qualitative data |
| 25 | Antibiotic prescribing practice and adherence to guidelines in primary care in the Cape Town Metro District, South Africa | Gasson, J;Blockman, M;Willems, B | 2018 | South African Medical Journal | 108 | 4 | Does not include original qualitative data |
| 26 | Patterns of prescription and drug dispensing | Karande, S.; Sankhe, P.; Kulkarni, M. | 2005 | Indian Journal of Pediatrics | 72 | 2 | Does not include original qualitative data |
| 27 | Realities of paediatric pharmacotherapy in the developing world | Hoppu, K.; Sri Ranganathan, S.; Dodoo, A. N. | 2011 | Archives of Disease in Childhood | 96 | 8 | Does not include original qualitative data |
| 28 | The impact of WHO essential medicines policies on inappropriate use of antibiotics | Holloway, K. A.; Rosella, L.; Henry, D. | 2016 | PLoS ONE | 11 | 3 | Does not include original qualitative data |
| 29 | WHO Essential Medicines Policies and Use in Developing and Transitional Countries: An Analysis of Reported Policy Implementation and Medicines Use Surveys | Holloway, K. A.; Henry, D. | 2014 | PLoS medicine | 11 | 9 | Does not include original qualitative data |
| 30 | Availability of essential medicines in Sudan | Elamin, Elfatih Ibrahim;Ibrahim, Mohamed Izham Mohamed;Yousif, MIM | 2010 | Sudanese Journal of Public Health | 5 | 1 | Does not include original qualitative data |
| 31 | Developing nations and the compulsory license: maximizing access to essential medicines while minimizing investment side effects | Bird, R. C. | 2009 | J Law Med Ethics | 37 | 2 | Does not include original qualitative data |
| 32 | Assessment of Pharmaceutical Services in a Southern Brazilian City | Bittencourt, R. A. S., M. P. T.; Guttier, M. C.; Miranda, F. F.; Bertoldi, A. D. | 2017 | Rev Bras Epidemiol | 20 | 2 | Does not include original qualitative data |
| 33 | Essential cancer medicines in the national lists of countries of the WHO South-East Asia Region: a descriptive assessment | Chivukula, M. V. T., Klara | 2018 | WHO South East Asia J Public Health | 7 | 2 | Does not include original qualitative data |
| 34 | ESMO International Consortium Study on the availability, out-of-pocket costs and accessibility of antineoplastic medicines in countries outside of Europe | Cherny, N. I.; Sullivan, R.; Torode, J.; Saar, M.; Eniu, A. | 2017 | Annals of Oncology | 28 | 11 | Does not include original qualitative data |
| 35 | Implementing the essential medicine concept in the country with the highest GDP per capita in the world | Cheraghali, A. M. | 2013 | East Mediterr Health J | 19 | 1 | Does not include original qualitative data |
| 36 | Four years of essential drugs' list in Nigeria | Adikwu, M. U. O., B. O. | 1991 | Soc Sci Med | 33 | 9 | Does not include original qualitative data |
| 37 | A baseline study of drug prescribing practices in a Nigerian military hospital | Adebayo, E. T. H., N. A. | 2009 | Niger J Clin Pract | 12 | 3 | Does not include original qualitative data |
| 38 | Pattern of prescription drug use in Nigerian army hospitals | Adebayo, E. T. H., N. A. | 2010 | Ann Afr Med | 9 | 3 | Does not include original qualitative data |
| 39 | Assessment of medicines use pattern using World Health Organization's Prescribing, Patient Care and Health facility indicators in selected health facilities in eastern Ethiopia | Bilal, A. I.; Osman, E. D.; Mulugeta, A. | 2016 | BMC Health Services Research | 16 |  | Does not include original qualitative data |
| 40 | Antibiotic stewardship: Factors influencing the choice and outcomes of antimicrobial therapy in a resource-limited, rural, public hospital in uMkhanyakude District, KwaZulu-Natal, South Africa: Pre-intervention phase | Abahamye, A. | 2016 | SA Pharmaceutical Journal | 83 | 8 | Does not include original qualitative data |
| 41 | Comparative efficacy and acceptability of first- and second-generation antidepressants in the acute treatment of major depression: a multiple treatments meta-analysis | Andrea, Cipriani; John, Geddes; Toshiaki, A. Furukawa; Georgia, Salanti; Corrado, Barbui; Stefan, Leucht; Guy, Goodwin; Eric, Ruhe; Sarah, Stockton; Julian, Higgins | 2012 |  |  |  | Does not include original qualitative data |
| 42 | Comparative efficacy and safety of intermediate-acting, long-acting and biosimilar insulins for type 1 diabetes mellitus: a systematic review and network meta-analysis | Andrea, Tricco; Areti-Angeliki, Veroniki; Huda, Ashoor; Jesmin, Antony; Sharon, Straus | 2017 |  |  |  | Does not include original qualitative data |
| 43 | New procedures for updating the model list of essential medicines | Anonymous, | 2002 | WHO Drug Information | 16 | 2 | Does not include original qualitative data |
| 44 | Assessment of current prescribing practices using World Health Organization core drug use and complementary indicators in selected rural community pharmacies in Southern India | Aravamuthan, A.; Arputhavanan, M.; Subramaniam, K.; Udaya Chander J, S. J. | 2016 | Journal of Pharmaceutical Policy and Practice | 10 | 1 | Does not include original qualitative data |
| 45 | Accessibility and use of essential medicines in health care: Current progress and challenges in India | Bansal, D.; Purohit, V. K. | 2013 | Journal of Pharmacology and Pharmacotherapeutics | 4 | 1 | Does not include original qualitative data |
| 46 | Selection of essential medicines for diabetes in low and middle income countries: A survey of 32 national essential medicines lists | Bazargani, Y. T.; De Boer, A.; Leufkens, H. G. M.; Mantel-Teeuwisse, A. K. | 2014 | PLoS ONE | 9 | 9 | Does not include original qualitative data |
| 47 | Essential medicines for COPD and asthma in low and middle-income countries | Bazargani, Y. T.; De Boer, A.; Leufkens, H. G. M.; Mantel-Teeuwisse, A. K. | 2014 | Thorax | 69 | 12 | Does not include original qualitative data |
| 48 | ESMO European Consortium Study on the availability, out-of-pocket costs and accessibility of antineoplastic medicines in Europe | Cherny, N. I.; Sullivan, R.; Torode, J.; Saar, M.; Eniu, A. | 2016 | Annals of Oncology | 27 | 8 | Does not include original qualitative data |
| 49 | Pharmacological and psychosocial management of mental, neurological and substance use disorders in low- and middle-income countries: issues and current strategies | de Jesus Mari, J.; Tofoli, L. F.; Noto, C.; Li, L. M.; Diehl, A.; Claudino, A. M.; Juruena, M. F. | 2013 | Drugs | 73 | 14 | Does not include original qualitative data |
| 50 | Presurgical Antibiotic Prophylaxis Pattern In An Indian Tertiary Care Teaching Hospital | Kaur, Rupinder; Salman, Mohd T.; Gupta, Narendra K.; Gupta, Uma; Ahmad, Ali; Verma, Vinod K. | 2016 |  |  |  | Does not include original qualitative data |
| 51 | Essential Medicines List Implementation Dynamics: A Case Study Using Brazilian Federal Medicines Expenditures | Magarinos-Torres, Rachel; Lynd, Larry David; Luz, Tatiana Chama Borges; Marques, Paulo Eduardo Potyguara Coutinho; Osorio-de-Castro, Claudia Garcia Serpa | 2017 | Basic & Clinical Pharmacology & Toxicology | 121 | 3 | Does not include original qualitative data |
| 52 | Are essential medicines in Malaysia accessible, affordable and available? | Saleh, K.; Ibrahim, M. I. M. | 2005 | Pharmacy World and Science | 27 | 6 | Does not include original qualitative data |
| 53 | A status survey on Xintian Central Township Health Center, Lintao County, Gansu Province | Shen, J. T.; Yang, X. Y.; Li, Y. P.; Li, H. H.; Fang, R.; Yi, J. L.; Han, J. X.; Hu, J. X.; Li, S.; Liu, Y. M.; Zhang, X. R.; Qin, H. W.; Pan, H. X.; Kang, X. Y.; Zhang, W. D.; Wang, X. L. | 2011 | Chinese Journal of Evidence-Based Medicine | 11 | 2 | Does not include original qualitative data |
| 54 | Current perspectives on China's national essential medicine system: Primary care provider and patient views Health policy, reform, governance and law | Song, Y.; Bian, Y.; Li, L. | 2016 | BMC Health Services Research | 16 | 1 | Does not include original qualitative data |
| 55 | Rational use effects of implementing an essential medicines list in West Bank, Palestinian Territories | Younis, M. Z.; Hamidi, S.; Forgione, D. A.; Hartmann, M. | 2009 | Expert Review of Pharmacoeconomics and Outcomes Research | 9 | 3 | Does not include original qualitative data |
| 56 | Understanding the Role and Use of Essential Medicines Lists | IMS Institute for Healthcare Informatics | 2015 |  |  |  | Does not include original qualitative data |
| **57** | Monitoring medicines use to support national medicines policy development and implementation in the Asia Pacific region | Roughead, E. E.; Lhazeen, K.; Socialine, E.; Bahri, S.; Park, B. J.; Holloway, K. | 2013 | WHO South East Asia J Public Health | 2 | 2 | Does not include original qualitative data |
| 1 | Patient access to anti-cancer medicines under public health insurance schemes in Thailand: A mixed methods study | Patikorn, C.; Taychakhoonavudh, S.; Thathong, T.; Anantachoti, P. | 2019 | Thai Journal of Pharmaceutical Sciences | 43 | 3 | Outside of the scope of WHO EML |
| 2 | Promoting access to cancer medicines in Mexico: Seguro Popular key policy components | Moye-Holz, D.; Dreser, A.; Gomez-Dantes, O.; Wirtz, V. J. | 2019 | 4th International Pharmaceutical Pricing and Reimbursement Information Conference, PPRI | 12 | Suppl 3 | Outside of the scope of WHO EML |
| 3 | Inflated medicine prices in Vietnam: A qualitative study | Nguyen, T. A.; Knight, R.; Mant, A.; Razee, H.; Brooks, G.; Dang, T. H.; Roughead, E. E. | 2017 | Health Policy and Planning | 32 | 5 | Outside of the scope of WHO EML |
| 4 | Current perspectives on China's national essential medicine system: primary care provider and patient views | Song, Y. B., Y.; Li, L. | 2016 | BMC Health Serv Res | 16 |  | Outside of the scope of WHO EML |
| 5 | A study on strategic planning and procurement of medicals in Uganda’s regional referral hospitals | Masembe, I. K. | 2016 | Journal of Public Health in Africa | 7 | 2 | Outside of the scope of WHO EML |
| 6 | Analyzing implementation dynamics using theory-driven evaluation principles: lessons learnt from a South African centralized chronic dispensing model | Magadzire, B. P. M., B.; Mathys, T.; Laing, R. O.; Ward, K. | 2017 | BMC Health Serv Res | 17 | Suppl 2 | Outside of the scope of WHO EML |
| 7 | Prescribing indicators in primary health care in Belo Horizonte, Brazil: associated factors | Lima, M. G.; Dutra, K. R.; Martins, U. C. M. | 2017 | International Journal of Clinical Pharmacy | 39 | 4 | Outside of the scope of WHO EML |
| 8 | Rationality of drug prescriptions in rural health centres in Burkina Faso | Krause, G. B., M.; Benzler, J.; Heinmuller, R.; Kaba, I.; Savadogo, M.; Siho, N.; Diesfeld, H. J. | 1999 | Health Policy Plan | 14 | 3 | Outside of the scope of WHO EML |
| 9 | Analysis on the utilization status of essential medicines in beijing's community health institutions | Zhang, F.; Li, S. C.; Yang, Y. | 2016 | Value in Health | 19 | 3 | Outside of the scope of WHO EML |
| 10 | Analysis on the utilization status of essential medicines in Beijing's community health institutions | Zhang, F.; Li, S. C. | 2016 | Value in Health | 19 | 7 | Outside of the scope of WHO EML |
| 11 | Reasons for physicians' tendency to irrational prescription of corticosteroids | Yousefi, N.; Majdzadeh, R.; Valadkhani, M.; Nedjat, S.; Mohammadi, H. | 2012 | Iranian Red Crescent Medical Journal | 14 | 11 | Outside of the scope of WHO EML |
| 12 | Making Drugs Affordable for the Poor in a Tertiary Care Hospital: A Case Study | Singh, O. P.; Bapna, J. S. | 2008 | Journal of Health Management | 10 | 2 | Outside of the scope of WHO EML |
| 13 | Evaluation of drug use indicators for non-communicable diseases in Pakistan | Riaz, H. G., B.; Bashir, S.; Hussain, S.; Mahmood, S.; Waseem, D.; Malik, F.; Raza, S. A. | 2016 | Acta Pol Pharm | 73 | 3 | Outside of the scope of WHO EML |
| 14 | Do national medicinal drug policies and essential drug programs improve drug use?: a review of experiences in developing countries | Ratanawijitrasin, S. S., S. B.; Weerasuriya, K. | 2001 | Soc Sci Med | 53 | 7 | Outside of the scope of WHO EML |
| 15 | Changing global essential medicines norms to improve access to AIDS treatment: lessons from Brazil | Nunn, A. F., E. D.; Gruskin, S. | 2009 | Glob Public Health | 4 | 2 | Outside of the scope of WHO EML |
| 16 | Prescribing and dispensing activities at the health facilities of a non-governmental organization | Hazra, A.; Tripathi, S. K.; Alam, M. S. | 2000 | National Medical Journal of India | 13 | 4 | Outside of the scope of WHO EML |
| 17 | Three years evaluation of drug shortages from educational pharmacies in Tehran | Gholami, K.; Kamalinia, G.; Attari, M. M. A.; Salamzadeh, J. | 2012 | Iranian Journal of Pharmaceutical Research | 11 | 2 | Outside of the scope of WHO EML |
| 18 | Availability of pediatric medicines and their perception among prescribers at a tertiary care teaching hospital | Desai, M.; Jain, K.; Shah, S.; Dikshit, R. K. | 2012 | Journal of Applied Pharmaceutical Science | 2 | 8 | Outside of the scope of WHO EML |
| 19 | Better medicines for children' within the Integrated Management of Childhood Illness framework: a qualitative inquiry in Uganda | Nsabagasani, X.; Ogwal-Okeng, J.; Hansen, E. H.; Mbonye, A.; Muyinda, H.; Ssengooba, F. | 2016 | Journal of Pharmaceutical Policy & Practice | 9 |  | Outside of the scope of WHO EML |
| 20 | The UN Commission on Life Saving Commodities 3 years on: global progress update and results of a multicountry assessment | Pronyk, P. M.; Nemser, B.; Maliqi, B.; Springstubb, N.; Sera, D.; Karimov, R.; Katwan, E.; Walter, B.; Bijleveld, P.; U. NCoLSC Technical Resource Teams; U. N. Agency Leads; U. NCoLSC Monitoring; Evaluation Advisory, Group | 2016 | The Lancet Global Health | 4 | 4 | Outside of the scope of WHO EML |
| 21 | Analysis of the pharmaceutical assistance cycle in Romelandia, Santa Catarina, Brazil | Prevedello, P.; Busato, M. A. | 2014 | Brazilian Journal of Pharmaceutical Sciences | 50 | 1 | Outside of the scope of WHO EML |
| 22 | Factors influencing the implementation of integrated management of childhood illness (IMCI) by healthcare workers at public health centers & dispensaries in Mwanza, Tanzania | Kiplagat, A.; Musto, R.; Mwizamholya, D.; Morona, D. | 2014 | BMC Public Health | 14 | 1 | Outside of the scope of WHO EML |
| 23 | The establishment and expansion of an innovative centre for rational pharmacotherapy--determinants and challenges | Kardakis, T. T., G.; Wettermark, B.; Brommels, M.; Godman, B.; Bastholm-Rahmner, P. | 2015 | Int J Health Plann Manage | 30 | 1 | Outside of the scope of WHO EML |
| 24 | Health-care providers’ preparedness for H1N1/09 influenza prevention and treatment in Dar es Salaam, Tanzania | Kamuhabwa, Appolinary;Chavda, Reena | 2011 | The Journal of Infection in Developing Countries | 6 | 3 | Outside of the scope of WHO EML |
| 25 | Trends and challenges toward integration of traditional medicine in formal health-care system: Historical perspectives and appraisal of education curricula in Sub-Sahara Africa | Innocent, E. | 2016 | Journal of Intercultural Ethnopharmacology | 5 | 3 | Outside of the scope of WHO EML |
| 26 | Primary care physicians report high trust in and usefulness of the Stockholm drug and therapeutic committee's list of recommended essential medicines (the 'Wise List') | Eriksen, J. O., M. L.; Vallin, M.; Juhasz-Haverinen, M.; Andersen-Karlsson, E.; Ateva, K.; Gustafsson, L. L.; Jirlow, M.; Bastholm-Rahmner, P. | 2018 | Eur J Clin Pharmacol | 74 | 1 | Outside of the scope of WHO EML |
| 27 | Technical issues and conservation conditions of medicines in the primary health care of the Brazilian Unified Health System | Costa, E. A. A., P. S.; Pereira, M. T.; Souto, A. C.; Souza, G. S.; Leite, S. N. | 2017 | Rev Saude Publica | 51 | suppl 2 | Outside of the scope of WHO EML |
| 28 | How to establish a successful revolving drug fund: the experience of Khartoum state in the Sudan | Ali, G. K. | 2009 | Bull World Health Organ | 87 | 2 | Outside of the scope of WHO EML |
| 29 | Assessment of policy and access to HIV prevention, care, and treatment services for men who have sex with men and for sex workers in Burkina Faso and Togo | Duvall, S.; Irani, L.; Compaore, C.; Sanon, P.; Bassonon, D.; Anato, S.; Agounke, J.; Nigobora, B.; MacInnis, R. | 2015 | Journal of Acquired Immune Deficiency Syndromes: JAIDS | 68 | Suppl 2 | Outside of the scope of WHO EML |
| 30 | Indian vaccine innovation: The case of Shantha Biotechnics | Chakma, J.; Masum, H.; Perampaladas, K.; Heys, J.; Singer, P. A. | 2011 | Globalization and health | 7 |  | Outside of the scope of WHO EML |
| 31 | Factors affecting learning and teaching for medicines supply management training in Pacific Island Countries--a realist review | Brown, A. N.; Ward-Panckhurst, L.; Cooper, G. | 2013 | Rural and remote health | 13 | 2 | Outside of the scope of WHO EML |
| 32 | Advancing the application of systems thinking in health: provider payment and service supply behaviour and incentives in the Ghana National Health Insurance Scheme--a systems approach | Agyepong, I. A. A., G. C.; Nonvignon, J.; Asenso-Boadi, F..; Aikins, M.; Arhinful, D. K. | 2014 | Health Res Policy Syst | 12 |  | Outside of the scope of WHO EML |
| 33 | Essential medicines for emergency care in Africa | Broccoli, Morgan C.; Pigoga, Jennifer L.; Nyirenda, Mulinda; Wallis, Lee; Calvello Hynes, Emilie J. | 2018 | Emerg Med J | 35 | 7 | Outside of the scope of WHO EML |
| 34 | Overview of Traditional Medicine in ECOWAS Member States | Busian,; KofiKasilo,; Ossy, M. J. | 2010 |  |  |  | Outside of the scope of WHO EML |
| 35 | Integrating palliative care into national health systems in Africa: a multi-country intervention study | Grant, L.; Downing, J.; Luyirika, E.; Murphy, M.; Namukwaya, L.; Kiyange, F.; Atieno, M.; Kemigisha-Ssali, E.; Hunt, J.; Snell, K.; Murray, S. A.; Leng, M. | 2017 | Journal of Global Health | 7 | 1 | Outside of the scope of WHO EML |
| 36 | Are national policies and programs for prevention and management of postpartum hemorrhage and preeclampsia adequate? A key informant survey in 37 countries | Smith, Jeffrey Michael; Currie, Sheena; Cannon, Tirza; Armbruster, Deborah; Perri, Julia | 2014 | Glob Health Sci Pract | 2 | 3 | Outside of the scope of WHO EML |
| 37 | Essential Drugs in Bangladesh and Role of Different Stake Holders - A qualitative study | Akter, Seikh Farid Uddin; Rashid, MA; Mazumder, SK; Jabbar, SA; Sultana, F; Rahman, MH; Zahedee, MNS %J Int J Sci Environ Technol | 2012 | Int J Sci Environ Technol | 1 | 5 | Outside of the scope of WHO EML |
| 38 | The use of Standard Treatment Guidelines and Essential Medicines List by professional nurses at primary healthcare clinics in the uMgungundlovu District in South Africa | Sooruth, Umritha Raj; Sibiya, Maureen Nokuthula; Sokhela, Dudu Gloria %J International Journal of Africa Nursing Sciences | 2015 |  | 3 |  | Outside of the scope of WHO EML |
| **39** | Essential drugs policy in three rural counties in China: what does a complexity lens add? | Xiao, Y.; Zhao, K.; Bishai, D. M.; Peters, D. H. | 2013 | Social Science & Medicine | 93 |  | Outside of the scope of WHO EML |
| 1 | Drawing lessons from the standard treatment guidelines and essential medicines list concept in south africa as the country moves towards national health insurance | Perumal-Pillay, V. A.; Suleman, F. | 2021 | South African Family Practice | 63 | 1 | Commentary, protocol, conf. abstract. |
| 2 | Assessing access to essential medicines list (EML) in the Republic of Moldova | Seicas, R.; Turcanu, G.; Bivol, S.; Carp, A. | 2019 | 4th International Pharmaceutical Pricing and Reimbursement Information Conference, PPRI | 12 | Suppl 3 | Commentary, protocol, conf. abstract. |
| 3 | Making the list: the role of essential medicines lists in reproductive health | Hutchings, Jane; Neroutsos, Keith; Donnelly, Kathleen | 2010 | International perspectives on sexual & reproductive health | 36 | 4 | Commentary, protocol, conf. abstract. |
| 4 | Essential medicines national professional officers planning and evaluation meeting : final report | N.A,; Kamuhabwa,; AppolinaryChavda,; Reena,; N.A,; N.A,; N.A,; Busian,; KofiKasilo,; Ossy, M. J. | 2012 |  |  |  | Commentary, protocol, conf. abstract. |
| 5 | The need for guidelines and the use of economic evidence in decision-making in Thailand: lessons learnt from the development of the national list of essential drugs | Wibulpolprasert, S. | 2008 | J Med Assoc Thai | 91 | Suppl 2 | Commentary, protocol, conf. abstract. |
| 6 | Implementing use of misoprostol for uterine evacuation in postabortion care (MPAC) in low resource settings | Osur, J. | 2012 | International Journal of Gynecology and Obstetrics | 3 |  | Commentary, protocol, conf. abstract. |
| 7 | Human rights approach to health policy: The case of essential medicines | Hogerzeil, H. V. | 2009 | Tropical Medicine and International Health | 2 |  | Commentary, protocol, conf. abstract. |
| 8 | Twenty-five years of essential medicines | Quick, J. D. H., H. V.; Velasquez, G.; Rago, L. | 2002 | Bull World Health Organ | 80 | 11 | Commentary, protocol, conf. abstract. |
| 9 | Innovation and the WHO's essential medicines list: giving credit where credit is due | Wertheimer, A. I. S., T. M. | 2007 | Res Social Adm Pharm | 3 | 1 | Commentary, protocol, conf. abstract. |
| 10 | Can the selection and use of essential medicines decrease inappropriate drug use? | Reidenberg, M. M. | 2009 | Clin Pharmacol Ther | 85 | 6 | Commentary, protocol, conf. abstract. |
| 11 | Analysis of essentiality of the medicines from the "popular pharmacy program in brazil" | Yamauti, S. M.; Barberato-Filho, S.; Lopes, L. C. | 2014 | Pharmacoepidemiology and Drug Safety | 1 |  | Commentary, protocol, conf. abstract. |
| 12 | Review of the existing legislations for blood systems of countries in the eastern Mediterranean region and a recommended model | Smit Sibinga, C.; Abdella, Y.; Konings, F. | 2018 | Vox Sanguinis | 113 | Suppl 1 | Commentary, protocol, conf. abstract. |
| 13 | Pricing and reimbursement (P&R) in bric countries | Shepelev, J.; Richard, L. | 2009 | Value in Health | 12 | 7 | Commentary, protocol, conf. abstract. |
| 14 | Malawi standard drug list; 1991; Malawi standard drug list; 1991: District Hospital drug list; Malawi standard drug list; 1991: Health Centres drug list; Overview of Traditional Medicine in ECOWAS Member States | N.A,; Busian,; KofiKasilo,; Ossy, M. J. | 2010 |  |  |  | Commentary, protocol, conf. abstract. |
| 15 | Results of parent support group and health professional surveys on knowledge about and access to essential medicines in low/middle income countries (LMICs) | Grynszpancholc, E.; Wernikowski, J.; Barr, R. | 2014 | Pediatric Blood and Cancer | 2) |  | Commentary, protocol, conf. abstract. |
| 16 | Health-Care Providers' Preparedness for H1N1/09 Influenza Prevention and Treatment in Dar es Salaam; Tanzania; Malawi standard drug list; 1991; Malawi standard drug list; 1991: District Hospital drug list; Malawi standard drug list; 1991: Health Centres drug list; Overview of Traditional Medicine in ECOWAS Member States | Kamuhabwa,; AppolinaryChavda,; Reena,; N.A,; N.A,; N.A,; Busian,; KofiKasilo,; Ossy, M. J. | 2012 |  |  |  | Commentary, protocol, conf. abstract. |
| 17 | Enhancing the Role of Traditional Medicine in Health Systems: A Strategy for the African Region; Essential medicines national professional officers planning and evaluation meeting : final | Kasilo, Ossy Muganga Julius; Nikiema, Jean Baptiste; Ota, Martin Matthew Okechukwu; Desta, Abayneh Tamir; Touré, Bokar; N.A,; Kamuhabwa,; AppolinaryChavda,; Reena,; N.A,; N.A,; N.A,; Busian,; KofiKasilo,; Ossy, M. J. | 2013 |  |  |  | Commentary, protocol, conf. abstract. |
| 18 | OS032. Pharmacotherapy for pre-eclampsia in low and middle income countries: An analysis of essential medicines lists (EMLS) | Lalani, S.; Firoz, T.; Magee, L. A.; Lowe, R.; Sawchuck, D.; Payne, B.; Gordon, R.; Vidler, M.; Dadelszen, Pv; Community, Level | 2012 | Pregnancy Hypertension | 2 | 3 | Commentary, protocol, conf. abstract. |
| 19 | Increasing access to diagnosis and treatments for hepatitis C (HCV) in resource limited settings (RLS), how should we move forwards? | Meyer-Aandrieux, I.; Roberts, T.; Gaspani, S.; Milani, B.; Cohn, J. | 2013 | Journal of Hepatology | 1 |  | Commentary, protocol, conf. abstract. |
| 20 | Availability of Essential Medicines in Sudan?; Enhancing the Role of Traditional Medicine in Health Systems: A Strategy for the African Region; | Mohamed, Ali; G, K.; Gasson, J.; Blockman, M.; Willems, B.; Elamin,; E. I. Ibrahim; M. I. M. Yousif; M. A, E.; Ossy, M. J. | 2009 |  |  |  | Commentary, protocol, conf. abstract. |
| 21 | Challenges to rational prescribing and use of essential medicines in India | Padhy, Biswa Mohan | 2014 |  |  |  | Commentary, protocol, conf. abstract. |
| 22 | How to implement national essential medicine list in shanghai: PROS & CONS | Hu, S.; Zhang, Y. B.; He, J. | 2010 | Value in Health | 13 | 3 | Commentary, protocol, conf. abstract. |
| 23 | Health technology assessment in developing the National List of Essential Medicines in Thailand | Thamlikitkul, V. | 2014 | J Med Assoc Thai | 97 | Suppl 5 | Commentary, protocol, conf. abstract. |
| 24 | Essential medicines by the world health organisation and their convenience in elderly patients | Fernandez, R.; Sanchez, D. S.; Del Rio Torres, H.; Jane, C. C. | 2017 | European Journal of Hospital Pharmacy | 24 | Suppl 1 | Commentary, protocol, conf. abstract. |
| 25 | Approaches to improving access to essential cancer medicines in the WHO South-East Asia Region | Chivukula, M. V. T., Klara | 2018 | WHO South East Asia J Public Health | 7 | 2 | Commentary, protocol, conf. abstract. |
| 26 | Access to and quality use of noncommunicable diseases medicines in Nepal | Bhuvan, K. C.; Heydon, S.; Norris, P. | 2015 | Journal of Pharmaceutical Policy and Practice | 8 | 1 | Commentary, protocol, conf. abstract. |
| 27 | Free essential medicines through public health facilities in Nepal: A qualitative study | Acharya, M.; Dahal, A. | 2016 | Value in Health | 19 (7) |  | Commentary, protocol, conf. abstract. |
| 28 | Global asthma network identifies gaps in essential asthma medicines | Asher, I.; Bissell, K.; Ellwood, P.; Ellwood, E.; Chiang, C. Y.; Marks, G.; El Sony, A.; Billo, N.; Perrin, C. | 2016 | European Respiratory Journal. Conference: European Respiratory Society Annual Congress | 48 | Suppl 60 | Commentary, protocol, conf. abstract. |
| 29 | Specific pharmacovigilance system in the use of the new therapeutic combination nifurtimox - Eflornithine in the treatment of second stage of gambiense human African trypanosomiasis | Franco, J. R.; Simarro, P. P.; Diarra, A.; Ruiz-Postigo, J. A.; Samo, M. | 2011 | Tropical Medicine and International Health | 1) |  | Commentary, protocol, conf. abstract. |
| **30** | Is WHO's essential medicines list contributing to rational medicines use in developing countries? | Gray, A.; Holloway, K. | 2010 | Basic and Clinical Pharmacology and Toxicology | 1) |  | Commentary, protocol, conf. abstract. |
| 1 | Pharmacotherapy for preeclampsia in low and middle income countries: an analysis of essential medicines lists | Lalani, S.; Firoz, T.; Magee, L. A.; Sawchuck, von Dadelszen, P.; Community Level Interventions for Pre-eclampsia Working, Group | 2013 | Journal of Obstetrics & Gynaecology Canada: JOGC | 35 | 3 | Refers to drugs as essential medicines, not NML policy |
| 2 | Antenatal corticosteroids for management of preterm birth: a multi-country analysis of health system bottlenecks and potential solutions | Liu, G.; Segre, J.; Gulmezoglu, A.; Mathai, M.; Smith, J. M.; Hermida, J.; Althabe, F.; Working Group for, U. N. Commission of Life Saving Commodities Antenatal Corticosteroids | 2015 | BMC Pregnancy & Childbirth | 15 Suppl 2 |  | Refers to drugs as essential medicines, not NML policy |
| 3 | Impact of China's essential medicines scheme and zero-mark-up policy on antibiotic prescriptions in county hospitals: a mixed methods study | Wei, X. Y., J.; Walley, J. D.; Zhang, Z.; Hicks, J. P.; Zhou, Y.; Sun, Q.; Zeng, J.; Lin, M. | 2017 | Trop Med Int Health | 22 | 9 | Refers to drugs as essential medicines, not NML policy |
| 4 | Comparison of essential drug list in a rural secondary care hospital in south India with Indian & World Health Organization list 2011 | Rao, S. G. T., D.; Zachariah, S.; Kannan, M. S.; Alvarez-Uria, G. | 2012 | Indian J Physiol Pharmacol | 56 | 4 | Refers to drugs as essential medicines, not NML policy |
| 5 | Availability of medicines in public sector health facilities of two North Indian States | Prinja, S.; Bahuguna, P.; Tripathy, J. P.; Kumar, R. | 2015 | BMC Pharmacology and Toxicology | 16 | 1 | Refers to drugs as essential medicines, not NML policy |
| 6 | Availability and use of magnesium sulphate at health care facilities in two selected districts of North Karnataka, India | Katageri, Geetanjali; Charantimath, Umesh; Joshi, Anjali; Vidler, Marianne; von Dadelszen, Peter | 2018 | Reprod Health | 15 | Suppl 1 | Refers to drugs as essential medicines, not NML policy |
| 7 | Situational analysis of antibiotic use and resistance in Ghana: policy and regulation | Yevutsey, S. K.; Buabeng, K. O.; Aikins, M.; Anto, B. P.; Biritwum, R. B.; Frimodt-Moller, N.; Gyansa-Lutterodt, M. | 2017 | BMC Public Health | 17 | 1 | Refers to drugs as essential medicines, not NML policy |
| 8 | Access to essential drugs in 11 Brazilian cities: a community-based evaluation and action method | Karnikowski, M. G. N., O. T.; Naves, J. O.; Silver, L. D. | 2004 | J Public Health Policy | 25 |  | Refers to drugs as essential medicines, not NML policy |
| 9 | Patterns of paediatric analgesic use in Africa: a systematic review | Madadi, P.; Enato, E. F.; Fulga, S.; Umeoduagu, C. C.; MacLeod, S. M.; Koren, G.; Einarson, T. R. | 2012 | Archives of Disease in Childhood | 97 | 12 | Refers to drugs as essential medicines, not NML policy |
| 10 | Pharmacotherapy for pre-eclampsia in low and middle income countries: An analysis of essential medicines lists (EMLS) | Lalani, S.; Firoz, T.; Magee, L. A.; Lowe, R.; Sawchuck, D.; Payne, B.; Gordon, R.; Vidler, M.; Von Dadelszen, P. | 2012 | Pregnancy Hypertension | 2 (3) |  | Refers to drugs as essential medicines, not NML policy |
| 11 | Barriers and facilitators to the quality use of essential medicines for maternal health in low-resource countries: An Ishikawa framework | Tran, Dan N.; Bero, Lisa A. | 2015 | J Glob Health | 5 | 1 | Refers to drugs as essential medicines, not NML policy |
| **12** | Availability of essential medicines in Ethiopia: an efficiency-equity trade-off? | Carasso, B. S. L., M.; Tesfaye, A.; Palmer, N. | 2009 | Trop Med Int Health | 14 | 11 | Refers to drugs as essential medicines, not NML policy |
| 1 | Agenda setting for essential medicines policy in sub-Saharan Africa: a retrospective policy analysis using Kingdon's multiple streams model | Mhazo, A. T.; Maponga, C. C. | 2021 | Health Research Policy and Systems | 19 | 1 | Not national implementation of WHO EML |
| **2** | Cochrane haematology reviews to inform world health organization's list of essential medicines on clinical value of high-priority cancer medicines | Piechotta, V.; Hirsch, C.; Ernst, M.; Goldkuhle, M.; Moja, L.; Skoetz, N. | 2021 | Hematological Oncology | 39 | Suppl 2 | Not national implementation of WHO EML |
| 1 | Selection Mode of Essential Medicine in South Africa and Its Enlightenment to China | Xiaoyu, Zhang; Jianzhou, Y. A. N.; Rong, Shao | 2020 | China Pharmacy |  | 12 | Not English |
| 2 | [Medicines prescribed to the elderly in a city in southern Brazil and the Municipal Medicines List] | Aziz, M. M. C., M. C.; d'Orsi, E. | 2012 | Cad Saude Publica | 28 | 1 | Not English |
| 3 | [The list of drugs in the Popular Pharmacy Program and the Brazilian National Pharmaceutical Care Policy] | Yamauti, S. M. B.-F., S.; Lopes, L. C. | 2015 | Cad Saude Publica | 31 | 8 | Not English |
| 4 | [Health sector reform and pharmaceutical policy in Peru] | Romero, C. P. | 2002 | Cad Saude Publica | 18 | 4 | Not English |
| 5 | [The Farmacia Popular do Brasil Program and aspects of public provision of medicines in Brazil] | Santos-Pinto Cdu, B. C. N., R.; Osorio-de-Castro, C. G. | 2011 | Cien Saude Colet | 16 | 6 | Not English |
| 6 | [Essential medicines and the selection process in management practices of pharmaceutical services in Brazilian states and municipalities] | Magarinos-Torres, R. P., V. L.; Oliveira, M. A.; Osorio-de-Castro, C. G. | 2014 | Cien Saude Colet | 19 | 9 | Not English |
| 7 | Essential medicines lists for children of WHO, India, South Africa, and EML of China: A comparative study | Liu, D.; Cheng, J.; Zhang, L. L.; Li, Y. P.; Zeng, L. N.; Zhang, C.; Gui, G. | 2017 | Journal of Evidence-based Medicine | 10 | 4 | Not English |
| 8 | [Adherence to essential medicines in cities from three Brazilian states] | Dal Pizzol Tda, S. T., D. J.; Heineck, I.; Ferreira, M. B. | 2010 | Cad Saude Publica | 26 | 4 | Not English |
| 9 | A status survey on disease constitution and cost of inpatients in xintian central township health center in Lintao County of Gansu Province, 2008-2010 | Li, H. H.; Yang, X. Y.; Li, Y. P.; Sheng, J. T.; Li, C. C. | 2011 | Chinese Journal of Evidence-Based Medicine | 11 | 2 | Not English |
| **10** | [Access to essential medicines in Africa: a global approach] | Millot, G. | 2006 | Med Trop (Mars) | 66 | 6 | Not English |
| **150** |  |  |  |  |  |  |  |
