# Supplementary material for: National adaptation and implementation of WHO Model List of Essential Medicines: A qualitative evidence synthesis
Source: PLoS Med. 2022 Mar 11;19(3):e1003944. doi: 10.1371/journal.pmed.1003944 (PMC8956172; doi:10.1371/journal.pmed.1003944)
Supplement: Attachment — Submitted filename: Response letter QES essential medicines list 20211213.docx [file pmed.1003944.s005.docx]

13 December 2021

Dear Dr. Beryne Odeny,

Thank you for considering our manuscript titled ‘National adaptation and implementation of the WHO Model List of Essential Medicines: A qualitative evidence synthesis’ (PMEDICINE-D-21-03040R1).

We have now revised the manuscript based on the feedback from the editors and reviewers. We found their comments helpful and hope you will agree with us that the revised manuscript is an improvement from our initial submission.

In response to the editors’ and reviewers’ suggestions we have made the following improvements to the manuscript.

- **Requests from the editors:**

*1)      Abstract:*

*a)      Please report your abstract according to PRISMA for abstracts, following the PLOS Medicine abstract structure (Background, Methods and Findings, Conclusions)*

*http://www.plosmedicine.org/article/info:doi/10.1371/journal.pmed.1001419* .

**Response:** We have updated the abstract accordingly.

*b)      Please combine the Methods and Results sections into one section, “Methods and findings”.*

**Response:** We have updated the abstract accordingly.

*c)      Please ensure that all numbers presented in the abstract are present and identical to numbers presented in the main manuscript text.*

**Response:** We have check that these numbers match.

*d)      In the last sentence of the Abstract Methods and Findings section, please describe the main limitation(s) of the study's methodology.*

**Response:** We have updated the abstract accordingly.

*2)      At this stage, we ask that you include a short, non-technical Author Summary of your research to make findings accessible to a wide audience that includes both scientists and non-scientists. The Author Summary should immediately follow the Abstract in your revised manuscript. This text is subject to editorial change and should be distinct from the scientific abstract. Please see our author guidelines for more information: https://journals.plos.org/plosmedicine/s/revising-your-manuscript#loc-author-summary*

**Response:** We have added a Summary of research following the Abstract.

*3)      Please submit a completed PRISMA checklist. We understand that not all items on the checklist will be directly relevant, and these can be marked as ‘not applicable’.*

*a)      The PRISMA guidelines provided at the EQUATOR site http://www.equator-network.org/reporting-guidelines/prisma/*

*b)      When completing the checklist, please use section and paragraph numbers, rather than page numbers.*

*c)      Please add the following statement, or similar, to the Methods: "This study is reported as per the Preferred Reporting Items for Systematic Reviews and Meta-Analyses (PRISMA) guideline (S1 Checklist)."*

**Response:** We have completed the PRISMA checklist and it has been submitted with this manuscript and have added the necessary statement to the Methods section of the manuscript.

*4)      In line with PLOS Medicine’s guidelines, please update your search to the present time and provide the beginning and end dates of your search.*

**Response:** We have updated the search and the review findings and 23 articles were included in the the review.

*5)      Please avoid assertions of primacy (e.g.," This is the first systematic review of…"). Please temper claims of primacy of results by stating, "to our knowledge" or something similar.*

**Response:** We have reviewed the manuscript and such statements have been revised.

*6)      References:*

*a)      Please reformat the citation style into PLOS Medicine's format. Please ensure there is no space between numbers in reference call outs within the main text, and the period is placed after the call out. For example, “…NLMs [7,8].”*

**Response:** The references have been reformatted to meet PLOS Medicine formatting.

*b)      Please ensure that journal name abbreviations consistently match those found in the National Center for Biotechnology Information (NCBI) databases. https://journals.plos.org/plosmedicine/s/submission-guidelines#loc-references.*

**Response:** The journal abbreviations have been updated to align with PLOS Medicine formatting.

*c)      Please update reference #48 or remove if it has not yet been published*

**Response:** The relevant reference has now been published and is updated accordingly.

*d)      Please provide weblinks and access dates for reference # 2, 3, 4 and 43. In references #4 ad 43 please update the author’s name to WHO.*

**Response:** These references have been updated.

*7)      Please remove the ‘Funding’, from the end of the main text. In the event of publication, this information will be published as metadata based on your responses to the submission form.*

**Response:** This has now been removed.

- **Reviewer #1:**

*It would be very useful if authors provided the list (as a supplementary document) of excluded papers with the rationale for the exclusion.*

**Response:** We have provided this, see supplementary file 1 for a list of full texts reviewed and reasons for exclusion.

*Pg 5. It would be nice if authors checked income status with the World Bank and clearly state it in e.g. Table 2. More elaborate discussion of findings in light of income status of the country in question would be welcome? If data are not available on how many participants were in particular studies, maybe to state that fact, as an asterix in the table?*

**Response:** We have updated Table 3 and added a World Bank country income classification to column 2.

*Pg 4., paragraph #3, "…, Two reviewers…" the letter T should not be capital.*

**Response:** This has been corrected.

- **Reviewer #2:**

*1. Why did the authors not include quantitative articles in the review?  The qualitative lessons would be a lot more impactful if readers understood if there was any empirical basis on which the lessons were based. On a more minor note, the authors provide only in the table a quick summary of the underlying basis on which the recommendations were made in the qualitative articles themselves; it would be useful for some of that information to be integrated into the text as well.*

**Response:** The main reason for not including quantitative articles was because quantitative articles, such as comparisons of the global and national list or studies of temporal trends in the availability of essential medicines, do not respond to the primary needs of our research question. Our focus was on understanding how the WHO EML is used in countries and to identify factors affecting adaptation and implementation of the essential medicines list. From our perspective, adaptation and implementation processes for the essential medicines list are social phenomenon that are well-suited to be examined with qualitative methods. Moreover, quantitative studies on the EML are not examining process-related aspects of adaptation and implementation. It would therefore not be meaningful to synthesize quantitative evidence together with empirical observations from qualitative studies.

*2. The authors appear to consider all of the articles as contributing equally to the recommendations, but the articles were published across 2 decades and across many different countries from HIC to LMIC. Is an article from 20 years ago conducted in possibly a much different local health environment relevant to recommendations today?  More background on the context in which the recommendations were made would be helpful -- i.e. how can policymakers in France consider lessons from a study from China if their implementation systems are very different?*

**Response:** Nineteen of the twenty-three included articles were published since 2013. We acknowledge that policy contexts are different and settings change over time, however as it is a global list and it is not certain whether the relevance of the findings or the lessons for policy makers are so different across these contexts. We note that our manuscript does not aim to produce definitive recommendations that policymakers should consider, but rather highlight issues about the implementation of the EML that need greater attention. In terms of the concern that older studies may be given too much weight, we have primarily highlighted issues that were supported by research findings from multiple studies, and have used examples identified in individual articles to support our findings that were made up of multiple studies. Finally, we have given greater attention to themes that are supported by qualitative evidence where we have moderate to high confidence in the findings, based on the CERQUAL assessment.

*Even on a more specific note, the authors assigned the articles to different levels of confidence (high->low) based on the features of the report, but then appeared not to use those assignments in deriving their recommendations, since they appear to equally rely on low confidence conclusions from citations #29 and #32 just as strongly as high-confidence citations. Some recognition that a recommendation is based on low-confidence insights would be helpful. The same comment refers to the different qualitative bases on which the conclusions in the articles are reached -- some are based on actual qualitative research, some just on document review -- which is more reliable?*

**Response:** We acknowledge that the previous version of the manuscript did not sufficiently highlight the reported confidence in the review findings. In practice, it has been integral to drafting the results as using the CERQual process greatly influenced the data analysis as we completed the four CERQual considerations (Adequacy, Coherence, Relevance, Methodological assessment).

Secondly, the new search and addition of four articles to the review increased the certainty of our findings, and therefore no findings are reported with low confidence.

To address the Reviewer #2’s comment, we have now added additional wording to the manuscript for those findings that reported the greatest confidence (review findings 6, 7, 19, 12, 15). In terms of the comment about the different qualitative bases, we did not make a distinction between whether the methods of the reported study were solely document review, or both interview and document review.

*3. Greater detail in the discussion should be provided on how to implement some of these ideas, perhaps identifying specific organizations or prioritizing recommendations with different levels of urgency.*

**Response:** We have revised the discussion and where appropriate had tried to mention implementation considerations in greater detail.

*4. Minor stylistic note: There are too many acronyms, which makes for complicated reading.*

**Response:** We have revised the manuscript and have tried to remove acronyms and use alternate wording, where possible

- **Reviewer #3:**

*# Value of WHO list of medicines*

*- The study has a laudatory perspective on the WHO EML, which offers a "trusted and objective reference list..." (Introduction). However, the WHO EML has several limitations and does not always list medicines that "best satisfy the priority health care needs of a country's population". In fact, the WHO EML omits many medicines for chronic medical conditions (e.g., diabetes, heart disease), which are routinely used in high-income countries. Therefore, the WHO EML might not be a reference document for many countries because it is not always grounded in the best evidence.*

**Response:** We believe the reviewer raised important critical points about the EML. We have edited to present a more neutral introduction of the WHO EML (“The World Health Organization (WHO) Model List of Essential Medicines (EML) offers a global evidence-informed reference list for countries to use in the adaptation of their national formulary, or national medicines list (NML)”).

Furthermore, we have introduced a new paragraph in the introduction that convey the message that it has been heavily debated whether the WHO EML represent an optimal reference point for national medicines policy (see p. 4/lines 25-52). The paragraph presents key issues such as tensions between selection of medicines and the affordability of drugs, lack of harmonization between the WHO EML and treatment guidelines and questions that have been raised about the selection process. However, notwithstanding these issues, we think a systematic assessment of the evidence base on adaptation and implementation processes has value precisely because the contested and debated nature of the global EML suggest that country-level processes are grappling with issues that merit greater attention. This point is underscored by the fact that there are many individual studies from a wide range of settings showing that the WHO EML is a starting point for national medicines selection processes, and this is probably an under-representation of the full experience countries have with the WHO EML.

*- In many countries (HICs, and LMICs; e.g., India), health policy and medicines fall under regional (e.g., state, provincial, etc.) jurisdiction. There may be a national EML and additional regional lists. In such instances, the regional list takes precedence over the national list.*

**Response:** We agree that this is an important point and have tried to highlight the challenges of national and regional medicine lists in this updated version of the manuscript.

*- Given the above points, I am not convinced that many countries are trying to adapt the WHO EML, which raises questions on the utility/value/contribution of this study. I am not confident that this is a major problem or barrier for many countries.*

**Response:** We agree that the merit in examining our research question could be debated a priori and that we shouldn’t take it as given that countries are using the WHO EML as a reference point (see also our response to a similar comment above). However, we think the fact that we identified many individual studies from a wide range of settings underscore the value of our research question, namely identifying factors affecting adaptation and implementation, with the goal of gaining a better understanding of what hampers or facilitates adapting global normative guidance on essential medicines at the country-level.

*# Data Source and Search Strategy*

*- The database search was led by an information specialist, which is a strong point.*

*- However, for this type of study, the grey literature is important because many policy documents might not be published in peer-reviewed journals.*

**Response:** Our review was primarily concerned with synthesizing empirical observations from qualitative studies that could be retrieved through a reproducible strategy, which we then also could subject to a confidence assessment using GRADE-CERQual. We agree that grey literature - such as reports from ministries, development funders or multilateral organizations - can be a valuable source of evidence about implementation processes. However, our assessment after including the peer-reviewed qualitative studies was that this body of knowledge was sufficient to respond to our research question. We have therefore not supplemented the evidence base with a systematic inquiry into the grey literature on this topic. We have acknowledged the lack of systematic inquiry into the grey literature in the limitation section, while explaining our rationale for not pursuing this step at length.

We agree that policy documents could be a valuable source if we were conducting a primary study of EML implementation in selected countries. However, policy documents do not by themselves provide sufficient insights into adaptation and implementation processes, which are issues one need to elicit experiences and views about from different types of stakeholders. In addition, the lack of transparency related to the methods used in writing policy documents would mean that it could be very difficult to complete a CERQual assessment of relevant findings identified in policy documents.

*- In conducting a literature search, I identified articles that were not included but seemed pertinent (e.g., Jarvis et al., CMAJ 2019; essential medicines in Canada). I was not part of this study and have no vested interest, but I was curious if this article would be relevant*.

**Response:** Thank you for bringing this to our attention. One reviewer reviewed all 1,567 unique titles again for this revision and we reassessed our decision to exclude Jarvis et al. (2019). We acknowledge that we were overly strict in this instance as the paper is not an example of a country that has implemented a national medicine list, however upon re-reading, it provides important perspectives from policy makers about the practicality of NMLs, especially for High-income settings. The paper has therefore been included in the review findings.

*- The Limitations mention that the search was done in July 2020, and "following the last update, two studies were added for data extraction". This means, following July 2020? How were these identified?*

**Response:** This has been reworded to reflect that the search was completed in 2021. The two articles added in 2020 were from the 2020 updated search, but we acknowledge that poor wording led to confusion.

*The authors should consider updating it. For literature sweeps, some journals accept if a single investigator screens articles and extracts data. The authors could consider this as a half-way solution.*

**Response:** We completed the search again and have added findings to the manuscript.

*# Scope of Review*

*- Table 2 outlines the types of studies included. It was not clear how the authors decided to combine studies on 'document reviews' and 'qualitative interviews'. If including document reviews, then it becomes more important to include the grey literature, as described above.*

**Response:** We deem primary studies that have ‘document reviews’ as the main source of evidence as one type of qualitative study and we did include such studies. Some qualitative studies relied primarily on interviews, while many relied on both document review and interviews.  However, we found that studies that included qualitative interviews and stakeholders’ views offered a crucial source for understanding adaptation and implementation processes, which then translated to greater emphasis in our data analysis.

*- It would be helpful if the study clarified if this list pertained to the adult only or adult and pediatric list of medicines (I apologize if I missed it).*

**Response:** The review did not differentiate between the two medicines lists and this has now been clarified in the manuscript.

*# Audience*

*- The study should specify the intended audience and target the Discussion accordingly. It is not clear if this is directed toward government leaders, policy makers, academics, etc. Consequently, the Results and Discussion appear to lack focus.*

**Response:** We have made the audience clearer by adding the following sentence to the conclusion. “The findings of this study may be valuable for national policy makers, especially those who are developing and implementing global normative guidance as the findings highlight areas that may require greater attention when improving NML adaptation and implementation”

*# Additional points*

*- Many strongly worded statements are without reference(s). Please review and provide references. For example "HICs may have more robust reimbursement processes..."*

**Response:** We have reviewed the manuscript again and added additional references.

*- Please review for typos*

**Response:** We have reviewed the manuscript again and corrected spelling and grammatical errors.

*- The Abstract should be revised. Were the 10 themes identified in all 18 studies (the current version suggests that). It would be helpful to summarize if one/few themes were found in all studies or a majority of the studies.*

**Response:** We have re-written the abstract to highlight three broad ideas that summarise the findings.

Please note that we have included Table 2 and 3 in the manuscript as originally submitted, so that the reviewers can easily see the track changes that we have made to the tables. Additionally, new clean tables have been uploaded to Editorial Manager.

Thank you again for considering our manuscript for publication.

Yours sincerely,

Ms. Elizabeth Peacocke, on behalf of the co-authors
